# Isolation of a gene cluster from *Armillaria gallica* for the synthesis of armillyl orsellinate–type sesquiterpenoids

**DOI:** 10.1007/s00253-020-11006-y

**Published:** 2020-11-16

**Authors:** Benedikt Engels, Uwe Heinig, Christopher McElroy, Reinhard Meusinger, Torsten Grothe, Marc Stadler, Stefan Jennewein

**Affiliations:** 1 Fraunhofer Institute for Molecular Biology and Applied Ecology, Forckenbeckstrasse 6, 52074 Aachen, Germany; 2 Present Address: Jennewein Biotechnologie GmbH, Maarweg 32, Rheinbreitbach, Germany; 3 Present Address: Department of Plant & Environmental Sciences, Weizmann Institute of Science, P.O. Box 26, 7610001 Rehovot, Israel; 4 Clemens Schöpf Institute of Organic Chemistry and Biochemistry, Technical University of Darmstadt, 64287 Darmstadt, Germany; 5 Mibelle Group Biochemistry, Bolimattstrasse 1, 5033 Buchs, Switzerland; 6 Department of Microbial Drugs, Helmholtz Centre for Infection Research, Inhoffenstrasse 7, 38124 Braunschweig, Germany

**Keywords:** Antibiotics, Chemotherapy, Drug discovery, Melleolide, Natural product biosynthesis, Terpenoid

## Abstract

**Abstract:**

Melleolides and armillyl orsellinates are protoilludene-type aryl esters that are synthesized exclusively by parasitic fungi of the globally distributed genus *Armillaria* (Agaricomycetes, Physalacriaceae). Several of these compounds show potent antimicrobial and cytotoxic activities, making them promising leads for the development of new antibiotics or drugs for the treatment of cancer. We recently cloned and characterized the *Armillaria gallica* gene *Pro1* encoding protoilludene synthase, a sesquiterpene cyclase catalyzing the pathway-committing step to all protoilludene-type aryl esters. Fungal enzymes representing secondary metabolic pathways are sometimes encoded by gene clusters, so we hypothesized that the missing steps in the pathway to melleolides and armillyl orsellinates might be identified by cloning the genes surrounding *Pro1*. Here we report the isolation of an *A. gallica* gene cluster encoding protoilludene synthase and four cytochrome P450 monooxygenases. Heterologous expression and functional analysis resulted in the identification of protoilludene-8α-hydroxylase, which catalyzes the first committed step in the armillyl orsellinate pathway. This confirms that ∆-6-protoilludene is a precursor for the synthesis of both melleolides and armillyl orsellinates, but the two pathways already branch at the level of the first oxygenation step. Our results provide insight into the synthesis of these valuable natural products and pave the way for their production by metabolic engineering.

**Key points:**

*• Protoilludene-type aryl esters are bioactive metabolites produced by Armillaria spp.*

*• The pathway-committing step to these compounds is catalyzed by protoilludene synthase.*

*• We characterized CYP-type enzymes in the cluster and identified novel intermediates.*

**Supplementary Information:**

The online version contains supplementary material available at 10.1007/s00253-020-11006-y.

## Introduction

Natural products provide a vast array of diverse chemical structures that can be exploited in the agricultural, food/feed, cosmetics, biofuels, textiles, and healthcare industries (Hyde et al. 2019). Terpenoids are by far the largest class of natural products, but only four are currently approved as antimicrobial drugs (tiamulin, valnemulin, retapamulin, and lefamulin), and these are all semisynthetic derivatives of the fungal antibiotic pleuromutilin (Mendes et al. 2016; Hunt 2019; Lin et al. 2019). Basidiomycetes provide a rich source of complex, structurally diverse, and bioactive sesquiterpenoids, and certain protoilludene-type sesquiterpene aryl esters produced by the genus *Armillaria* (class Agaricomycetes, family Physalacriaceae) are promising leads for the development of new drugs. There are ~ 130 *Armillaria* species, most of which are root pathogens that attack hardwood trees, conifers, fruit trees, and grapevines, thus threatening both timber and agronomic plantations (Kile et al. 1994; Baumgartner et al. 2011; Coetzee et al. 2018). Most *Armillaria* species are facultative necrotrophs, with parasitic and saprotrophic phases (Rishbeth 1985). During the parasitic phase, they colonize the cambium and kill the root tissue, which then provides nutrition during the saprotrophic phase. *Armillaria* mycelia can persist for months to years in residual root tissue and serve as an inoculum for further infections. The synthesis of protoilludene-type sesquiterpene aryl esters may help to prevent colonization by other saprotrophic fungi, thus avoiding competition for resources.

**Fig. 1:**
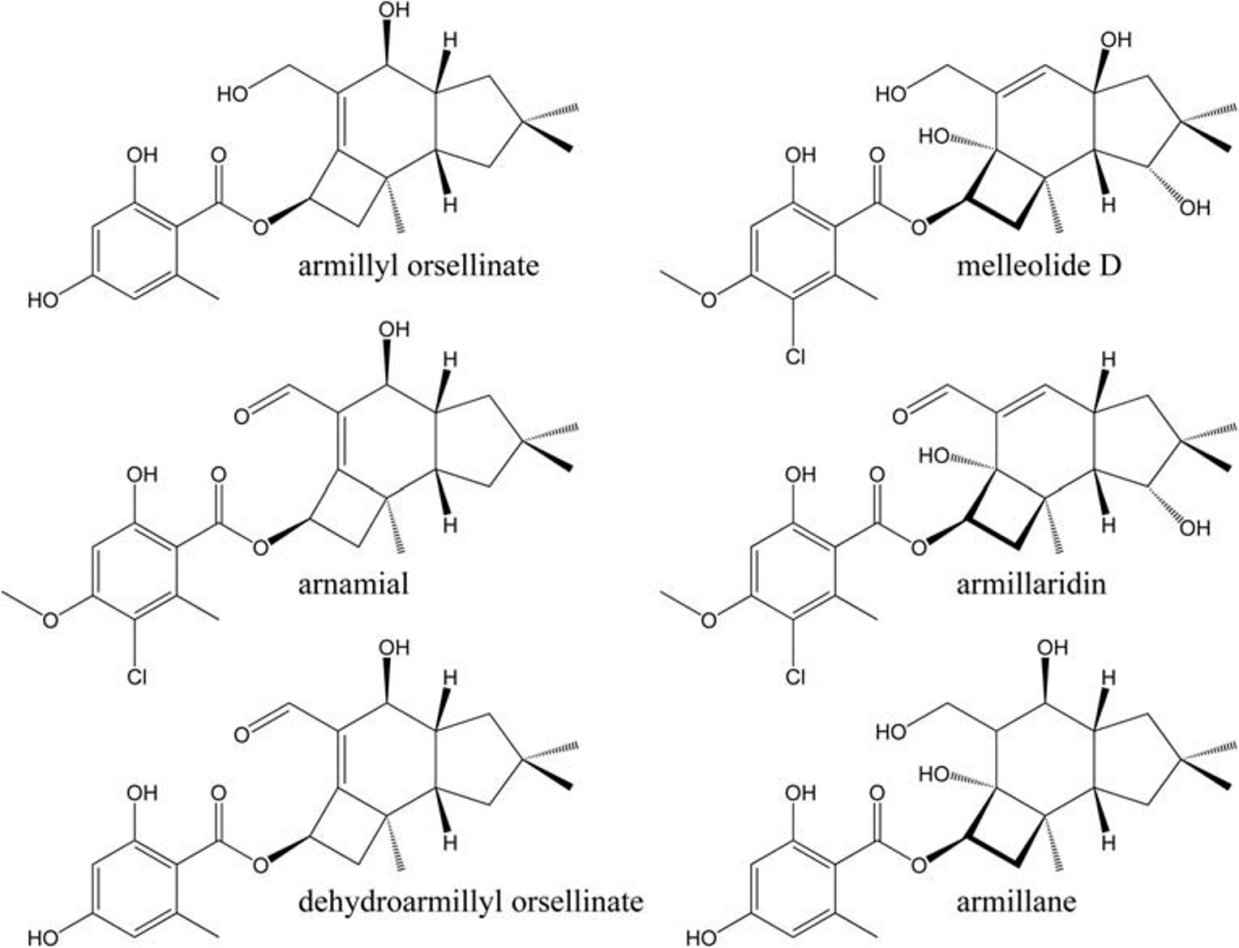
Selected armillyl orsellinate and melleolide structures produced by *Armillaria gallica* FU02472. The structures differ in the presence/absence or position of the double bond in the six-ring system, the number and position of hydroxyl groups, and modifications of the orsellinic acid side chain such as halogenation

**Fig. 2:**
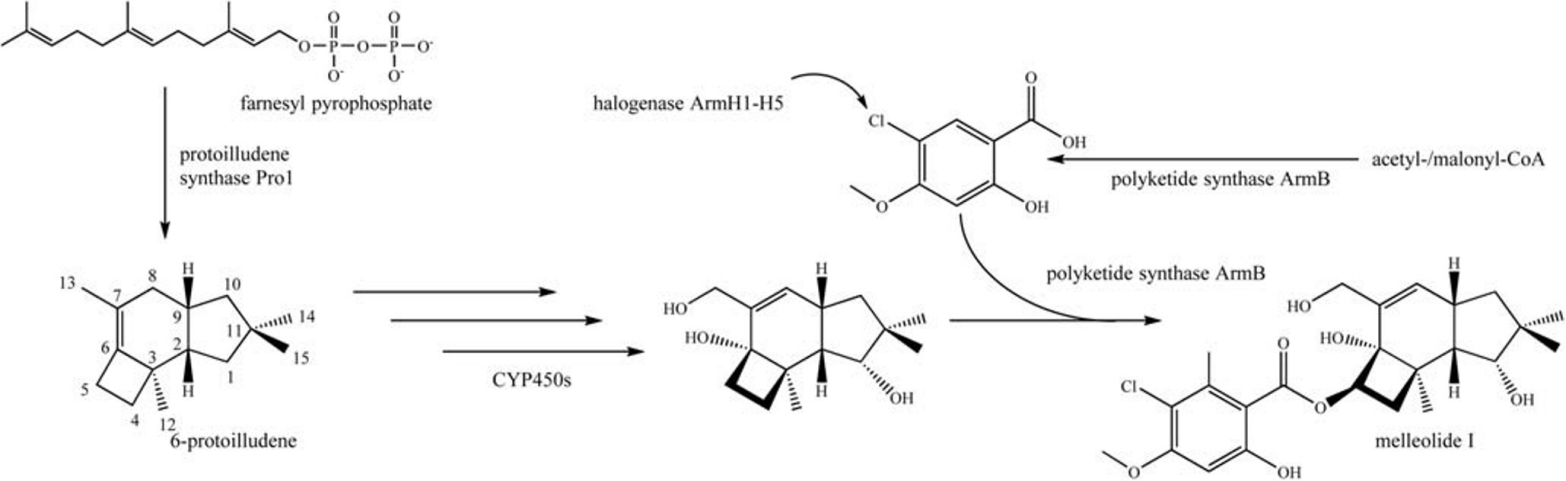
The melleolide I biosynthesis pathway. The pathway begins with the cyclization of farnesyl diphosphate to 6-protoilludene (Engels et al. 2011) and then oxygenation catalyzed by cytochrome P450 monooxygenases. The final step is the attachment of an orsellinic acid, produced, and transferred by the polyketide synthase ArmB (Lackner et al. 2013). Subsequent chlorination of the side chain is carried out by halogenases ArmH1–ArmH5 (Wick et al. 2016). In contrast to the main metabolite melleolide I, the structures of armillyl orsellinate and armillane (Bohnert et al. 2011) differ in the presence/absence or position of the double bond in the six-ring system and the number and position of hydroxyl groups

*Armillaria* species produce three classes of protoilludene-type aryl esters with different double bond localizations and functionalization patterns (Fig. 1), namely armillyl orsellinates (∆6–7), melleolides (∆7–8), and armillanes (absence of double bond within 6 ring) (Donnelly et al. 1982, Midland et al. 1982, Donnelly and Hutchinson 1990, Abraham 2001, Bohnert et al. 2014a, Dorfer et al. 2019a, b). For clarity, all carbon numbering is according to IUPAC standard. More than 70 armillyl orsellinate and melleolide structures have been reported, making them one of the most diverse fungal natural products discovered thus far (Misiek and Hoffmeister 2012; Dorfer et al. 2019a, b). They display a range of useful bioactivities, and mechanisms of action have been solved for some of the structures. For example, the fungicidal activity of arnamial and dehydroarmillyl orsellinate involves the inhibition of elongation factor 2 to block protein synthesis (Dorfer et al. 2019a, b), whereas the cytotoxicity of many of the structures reflects their ability to induce apoptosis (Bohnert et al. 2014a; Bohnert et al. 2014b; Li et al. 2016). Protoilludene-type aryl esters are synthesized by the esterification of protoilludanol alcohol and aromatic precursors derived from orsellinic acid, and this requires the prior cyclization of the universal sesquiterpene precursor farnesyl diphosphate to produce protoilludene (Engels et al. 2011). The protoilludene ring system then undergoes several hydroxylation reactions, most notably at the C6 position. C6 hydroxylation is thought to occur in combination with an allylic transposition of the C6-C7 double bond to the C7-C8 configuration found in melleolide-type products, presumably originating from the intermediate 6-protoilludene, which has been isolated from *Fomitopsis insularis* (Nozoe et al. 1977; Engels et al. 2011). Intriguingly, relocating the double bond to the C7-C8 position was found to abolish fungicidal activity (Misiek and Hoffmeister 2012; Bohnert et al. 2014a), but the position of the double bond does not influence the cytotoxicity of these compounds against human cancer cell lines. Furthermore, C-13 aldehyde melleolides show greater cytotoxicity than reduced melleolides (Bohnert et al. 2014a; Dorfer et al. 2019a, b). One of the likely final steps is the esterification of the Protoilludanol alcohols at the C5 hydroxyl position with polyketide orsellinic acid derivatives (Fig. 2) (Lackner et al. 2013). Although orsellinic acid and its derivatives are synthesized by many microorganisms, and protoilludanes are widespread among the Basidiomycetes, only the genus *Armillaria* is thought to couple these compounds.

Several isolated armillyl orsellinates and melleolides contain chlorinated orsellinic acid derivatives. The synthesis of the orsellinic side chain is catalyzed by the polyketide synthase ArmB, which was identified via elegant substrate feeding experiments (Lackner et al. 2013). Importantly, ArmB shows cross-coupling activity, making it a crucial step in the synthesis of melleolides, armillyl orsellinates, and armillanes (Lackner et al. 2013). Five FAD-dependent halogenases responsible for the C6′ chlorination of the orsellinic acid side chain have been cloned from *Armillaria mellea* (Wick et al. 2016). The missing steps in the biosynthesis of the armillyl orsellinates, armillanes, and melleolides are currently under investigation. Recently, we cloned and characterized a single-copy gene from *Armillaria gallica* encoding protoilludene synthase, which catalyzes the pathway-committing step to all armillyl orsellinates and melleolides (Engels et al. 2011). Interestingly, other terpene synthases have also been identified and well characterized from other basidiomycetes species; Omp6, a sesquiterpene synthase isolated from *Omphalotus olearius*, was also found to catalyze the conversion of (*E,E*) farnesyl diphosphate into 6-protoilludene (Quin et al. 2013). Additionally, it has been demonstrated that the product specificity of terpene synthases can be altered, even with single amino acid substitutions, for example, a His309Ala mutation within the pentalenene synthase led to the accumulation of 6-protoilludene (Segura et al. 2003).

The enzymes needed to produce several fungal secondary metabolites are encoded by gene clusters, including pleuromutilin produced by *Clitopilus pseudopinsitus* and *Clitopilus passeckerianus* (Bailey et al. 2016; Alberti et al. 2017; Yamane et al. 2017; Lin et al. 2019), erinacine diterpenoids produced by *Hericium erinaceus* (Yang et al. 2017), and bovistol and pasteurestin C produced by *Cyclocybe aegerita* (Surup et al. 2019). We therefore tested the hypothesis that the biosynthesis of protoilludene-type sesquiterpenoids in *A. gallica* may also involve a gene cluster and that the isolation of such a cluster would allow the characterization of the corresponding enzymes.

## Materials and methods

### Strains and growth conditions

*Armillaria gallica* strain FU02472 was cultivated as previously described (Engels et al. 2011); strain can be requested from authors. *Saccharomyces cerevisiae* strain CEN.PK2-1C (MATa, ura3-52, trp1-289, Leu2-3_112, his3∆1, MAL2-8^c^, SUC2) was obtained from the EUROpean *Saccharomyces cerevisiae* Archive for Functional analysis (EUROSCARF), University of Frankfurt, Frankfurt am Main, Germany, and maintained on SC minimal medium with agar (Engels et al. 2008). Buffered YP medium (2% w/v tryptone, 1% w/v yeast extract and 50 mM MES, pH 5.5) was supplemented with 2% w/v glucose for batch cultivation or 2% w/v galactose for fed-batch fermentation to induce gene expression driven by the *GAL1* promoter. *S. cerevisiae* was transformed using the lithium acetate method (Amberg et al. 2005) with selection as previously described (Engels et al. 2008).

### Genomic DNA isolation and library construction

*Armillaria gallica* genomic DNA was isolated using the cetyltrimethylammonium bromide method (Murray and Thompson 1980). A genomic library was constructed using the λDASH II/*Bam*HI vector kit with Gigapack III Gold packaging extracts according to the manufacturer’s guidelines (Stratagene, Heidelberg, Germany). The *A. gallica* genome walking library was constructed using the GenomeWalker Universal Kit according to the manufacturer’s protocol (Clontech, Saint-Germain-en-Laye, France). The amplification primers and PCR method are listed in the supplementary materials in Table S1.

### Library screening, genome walking, and sequencing

Phage plaques were transferred to charged nylon membranes (GE Healthcare, Little Chalfont, UK) and screened with ^32^P-labeled nucleic acid probes prepared using the DecaLabel kit (Thermo Fisher Scientific, Waltham, MA, USA). For pre-hybridization and hybridization, the membranes were incubated in RotiHybrid Quick buffer (Carl Roth, Karlsruhe, Germany) supplemented with 0.1 mg/mL salmon sperm DNA. The membranes were hybridized for 15 h at 65 °C and washed four times with RotiHybrid Quick buffer diluted in progressively larger volumes of water (1:2 dilution for 30 min, 1:5 dilution for 30 min, and 1:10 dilution for 2 × 10 min). The membranes were exposed to X-ray film at − 80 °C for 4 days. Isolated positive cosmids were sequenced by primer walking, and the inserts were amplified and subcloned for DNA sequencing using the Zero Blunt Cloning Kit (Invitrogen, Karlsruhe, Germany).

### Heterologous expression and functional analysis of cytochrome P450 monooxygenases

Four *A. gallica* cytochrome P450 monooxygenase (CYP) genes were cloned using the Gateway system and transferred to the galactose-inducible yeast expression vector pYES-DEST52 (Invitrogen). The enzymes were expressed in the haploid yeast strain CEN.PK2-1C, which was co-transformed with expression vector pCM183 (Gari et al. 1997) carrying the *Taxus chinensis* NADPH:cytochrome P450 reductase (*Tc*CPR) gene (Jennewein et al. 2005). Microsomal fractions were prepared from ~ 5 g (wet weight) of galactose-induced recombinant yeast biomass. Following the generation of spheroplasts and their disruption using glass beads, the resulting crude extract was clarified by centrifugation (4000×g, 4 °C, 10 min) and endoplasmic reticulum (ER) membranes were harvested from the supernatant by ultracentrifugation (105,000×*g*, 10 °C, 3 h). For functional analysis, protein extracts were analyzed by SDS-PAGE (Laemmli 1970; Fairbanks et al. 1971) and western blotting (Towbin et al. 1979). For functional testing, 50 mL YPG (2% galactose) cultures were cultivated in baffled shake flasks overnight at 28 °C overnight at 300 rpm; prior to substrate feeding, the cells were pelleted by centrifugation (3000×*g*, 5 min) and re-suspended with 5 mL of fresh YPG medium and incubated for an additional 4 h. After feeding with 6-protoilludene substrate (radioactive or otherwise), the 5-mL cultures were incubated overnight within a rotary shaker. Galactose-induced yeast cells co-expressing individual *A. gallica* CYP genes and *Tc*CPR (Jennewein et al. 2005) were incubated with either non-radioactive 6-protoilludene or [^3^H]-6-protoilludene produced from in vitro catalysis reactions using protein lysate originating from *Escherichia coli* BL21(DE3) CodonPlus cells transformed with the pDEST14::*Pro1*HIS6 vector, as previously described in Engels et al. (2011). After overnight incubation, 1 mL of saturated NaCl solution was added to the induced yeast cultures and the suspension was extracted three times with 5 mL *n*-pentane. The organic fractions were pooled and concentrated for analysis by radiolabeling thin layer chromatography (radio-TLC) and gas chromatography mass spectrometry (GC/MS) as previously described (Engels et al. 2011).

### GC/MS analysis

GC/MS analysis was carried out using a GC-QP2010S gas chromatograph quadrupole mass spectrometer (Shimadzu, Duisburg, Germany) equipped with an RxiTM-5 ms column (30 m long, 0.25 mm internal diameter; Restek, Bad Homburg, Germany). The samples were analyzed by heating to 80 °C for 2 min, followed by a heating rate of 15 °C/min to 300 °C, and a hold for 4 min at 300 °C. Sample fragmentation was induced by ionization at 1 keV; sample volume was 1 μL of solvent extracts unless otherwise stated.

### Biosynthesis of hydroxyprotoilludene in *S. cerevisiae*

The *S. cerevisiae* CEN.PK2-1C strain co-expressing CYP-Arm3 and *Tc*CPR was transformed with expression vector pRS315, in which a truncated version of yeast HMG-CoA reductase 1 (Dimster-Denk et al. 1994) was expressed under the control of the *PGK* promoter and *CYC1* transcriptional terminator. The *A. gallica* protoilludene synthase gene *Pro1* was cloned in vector pRS423 (complementing the histidine auxotrophy of CEN.PK2-1C) and was expressed under the control of the *GAL1* promoter and *CYC1* transcriptional terminator (both from vector pYES-DEST52).

### Fermentation of the hydroxyprotoilludene-producing *S. cerevisiae* strain

The *S. cerevisiae* production strain was cultivated in a 3.7-L fermenter (Bioengineering AG, Wald, Switzerland). The fermentation process consisted of two batch phases. In the first phase, 1.7 L of YPD medium was added to the fermenter vessel and sterilized in situ. The fermenter was then inoculated with 300 mL of a yeast culture in SD selection medium and incubated at 30 °C, with a rotor speed of 750 rpm. The pH of the medium was maintained at pH 5.5 by adding 0.5 M NaOH, and compressed air was bubbled through the medium at the maximum flow rate of 3.3 L/min. The dissolved O_2_ content was recorded using a KLF series pO_2_ electrode (Bioengineering AG). The second batch phase began when hunger signals were detected by the pO_2_ electrode, indicating that the glucose from the first batch had been metabolized (OD_600nm_ = 130–170). The temperature was reduced to 26 °C, the rotor speed was increased to 1500 rpm, and the pH was set to 6.5. Four hundred grams per liter galactose and 10× YP medium were incrementally added until 1 L of galactose medium had been added. When changes in pH and dissolved O_2_ indicated the onset of starvation, fermentation was terminated and the cells pelleted by centrifugation (3000×*g*, 4 °C, 5 min). The culture supernatant was extracted at 4 °C with silica C_18_ reversed phase powder, followed by filtration, lyophilization, and extraction with *n*-pentane using a Soxhlet apparatus. The crude extract was concentrated under vacuum using a rotary evaporator before being purified by chromatography on a 35-cm long, 40 g spherical silica gel 60 column of 0.020–0.045-mm particle size (Fluka) equilibrated with methylene chloride using 8, 10 mL methylene chloride fractions, followed by another 8 fractions using diethyl ether as elution solvent. The fractions were analyzed in parallel using the GC-MS, with the diethyl ether fractions containing the hydroxyprotoilludene product. The diethyl ether fractions were pooled and concentrated before being loaded onto another silica column (10 g, chloropropyl-functionalized silica gel) was eluted with 70% methylene chloride and 30% methanol. Two milliliters of fractions was collected and analyzed via GC-MS and TLC on silica gel 60 plates using 5% sulfuric acid in ethanol as staining solution. Spots became visible after heating, indicating that the hydroxyprotoilludene could be purified from another compound, undetectable with the GC-MS analysis method. Fractions containing the hydroxyprotoilludene were pooled and the solvents evaporated prior to purification via HPLC (LC-20A prominence, Shimadzu) on a Gemini 5u C18 110A semi-preparative column with a flow rate of 5 mL/min in a linear gradient from 70% methanol-water to 100% methanol using SPD-M20A photodiode array for detection and a FRC-10A for the collection of 2.5 mL fractions. The fractions were analyzed via GC-MS to confirm the fractions containing the hydroxyprotoilludene. Subsequently, the entire extract was likewise purified, and the hydroxyprotoilludene fractions pooled, the methanol distilled by rotary evaporation and the water were removed via anhydrous MgSO_4_. Finally, a mixture of MgSO_4_ and spherical silica gel was added to the water phase and the resulting dried powder transferred to a glass column, which was eluted with methylene chloride, with final purity analyzed via GC-MS, which indicated that the sample was pure enough for subsequent NMR analysis. After three purification steps, the fermentation broth yielded ~ 40 mg of pure product for further characterization by nuclear magnetic resonance (NMR) spectroscopy.

### NMR spectroscopy

All NMR spectra were recorded on a DRX 500-MHz NMR spectrometer (Bruker, Billerica, MA, USA) using a 5-mm BBO probe with a z-gradient. Hydroxyprotoilludene samples were dissolved in deuterated dichloromethane to a final concentration of ~ 50 mM and were calibrated against the solvent signals at 5.32 and 54.00 ppm. ^13^C-NMR spectra was acquired by conducting 1000 scans with a 30° pulse and a delay of 2 s. ^13^C-DEPT-135 spectra was also acquired by conducting 400 scans. All 2D spectra were acquired using Bruker standard pulse programs. The DQF-COSY and gs-NOESY spectra were measured in 512 increments, with two or four scans per increment, respectively. A mixing time of 600 ms was used for the acquisition of the NOESY spectrum. For heteronuclear correlation, the gs-HSQC and gs-HMBC spectra were measured in 512 increments, with four or eight scans per increment, respectively. The spectra were optimized for ^1^J and ^*n*^J couplings of 140 and 8 Hz, respectively. The data were processed using TopSpin v1.3 (Bruker) and MestReNova v8.0 (MestreLab Research, Santiago de Compostela, Spain).

### Access to sequence data

The sequences in this paper have been uploaded to GenBank (accession numbers MT277003).

## Results

### Identification of a biosynthetic gene cluster by phage library screening and genome walking

The enzymes involved in certain fungal secondary metabolic pathways are encoded by tightly linked gene clusters. Having recently discovered the single-copy *A. gallica Pro1* gene encoding protoilludene synthase (Engels et al. 2011), we hypothesized that other genes required for the synthesis of protoilludene-type aryl esters might be clustered with this gene. To address this hypothesis, we constructed an *A. gallica* genomic DNA library and used the previously isolated *Pro1* sequence as a probe to screen 3.5 × 10^4^ plaques with an average insert size of 20 kb. The size of the *A. gallica* genome is ~ 85 Mb (Sipos et al. 2017), but this was not yet known when we carried out the screen and we assumed a genome size of ~ 50 Mb based on the published sequences of the basidiomycetes *Laccaria bicolor* and *Coprinopsis cinerea*, with genome sizes of 65 and 36 Mb, respectively (Martin et al. 2008; Stajich et al. 2010). However, our screen was designed to achieve ~ 14-fold coverage, so the larger physical size of the *A. gallica* genome did not have a detrimental effect. The first round of screening yielded 17 candidates. These resolved in the second round to nine positive plaques containing genomic sequences that hybridized with the *Pro1* probe. Two of these plaques were used to isolate cosmid clones. Restriction digestion of the cosmid DNA produced identical fragments, which led to the conclusion that both cosmid inserts were the same. The ~ 23-kb insert of one of the cosmids (cosmid 5.1) was then sequenced by primer walking (using the primers in supplementary materials), which revealed the previously isolated *Pro1* gene and three sequences similar to cytochrome P450-dependent monooxygenases (CYPs), one of which appeared to be a partial sequence. In addition, the ~ 23 kb fragment contained two sequences with unknown functions (marked as hypothetical proteins) and one sequence homologous to glucose-methanol-choline (GMC) oxidoreductases.

**Fig. 3:**
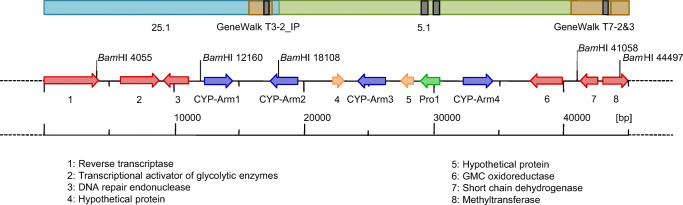
Map of the melleolide gene cluster. Schematic representation of the terpene biosynthesis part of the melleolide gene cluster obtained by screening a genomic DNA library with probes matching the protoilludene synthase coding region and untranslated regions (black bars). The cluster contains the protoilludene synthase gene (green arrow), four genes encoding cytochrome P450 monooxygenases (CYP, blue arrows), and two genes of unknown function (hypothetical proteins). Additional information for probe design was obtained by sequencing Genome Walker DNA fragments (brown bars), leading to the identification of cosmids 25.1 and 5.1

Protoilludene-type aryl esters are oxygenated at up to eight positions, so the identification of three similar CYP genes closely associated with *Pro1* provided strong supporting evidence for the presence of a biosynthetic gene cluster in *A. gallica*. We constructed a genome walking library by ligating DNA adaptors to restriction fragments of *A. gallica* genomic DNA and used the resulting PCR products to complete the partial CYP gene. Part of the newly identified sequence was then used to screen 1.7 × 10^4^ additional plaques in the original genomic library, resulting in the identification of a single positive clone that was confirmed in the second screening round. Sequencing the cosmid insert revealed a 16.27-kb genomic fragment containing an additional CYP gene (later named *CYP-Arm1*, with the others designated as *CYP-Arm2*, *CYP-Arm3*, and *CYP-Arm4*) as well as three putative genes with homology to a DNA repair endonuclease, a transcriptional activator, and a reverse transcriptase. Primer walking downstream of the cosmid 5.1 sequence revealed further genes that have yet to be investigated in the context of melleolide, armillyl orsellinate, and armillane biosynthesis (Fig. 3).

### Amplification of CYP genes in an *A. gallica* cDNA library

**Fig. 4:**
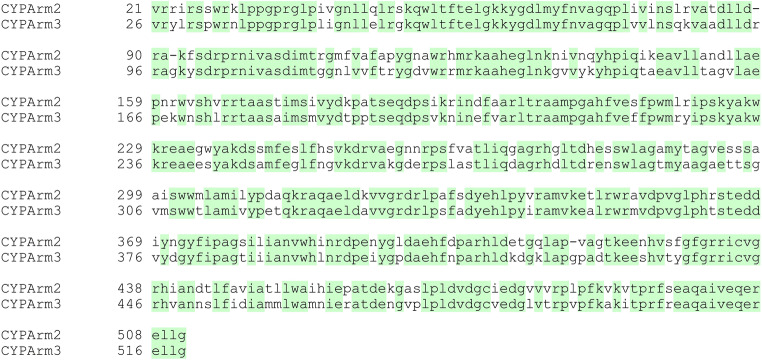
Alignment of the protein sequence shared between CYP-Arm2 and CYP-Arm3. The CYP-Arm3 sequence is eight residues longer than CYP-Arm2 and only the shared region is aligned, which introduces three single-residue gaps (−) to preserve the alignment; hence, five residues from CYP-Arm3 are missing. The two sequences share 75% identity when only the conserved sequence is considered

The amplified genomic DNA fragments were sequenced, revealing that all four CYP genes contained more than 10 exons (Fig. S1). The 2158-bp *CYP-Arm1* gene included 11 exons ranging in size from 38 to 537 bp and encoded a mature protein with 538 amino acids. The *CYP-Arm2* and *CYP-Arm3* genes (2122 and 2133 bp, respectively) were similar in structure, both featuring 12 exons, and encoded mature proteins with 513 and 521 amino acids, respectively. The 2272-bp *CYP-Arm4* gene included 15 exons ranging in size from 4 to 190 bp and encoded a mature protein with 509 amino acids. Global alignments of the protein sequences revealed 72% identity between CYP-Arm2 and CYP-Arm3 but only 27–32% identify for other comparisons. A pairwise comparison of the common sequence shared between CYP-Arm2 and CYP-Arm3 indicated that 75% identify within this region (Fig. 4).

Primers based on the genomic sequences were used to isolate corresponding cDNAs from an *A. gallica* cDNA library by PCR. Interestingly, a significant proportion of the cDNA clones still contained introns. The presence of introns was aided greatly with the use of the FGENESH tool (Salamov and Solovyev 2000); however, manual curation was still required as introns were present within some amplified cDNA sequences, due to faulty splicing. Despites this, full-length cDNA clones were obtained for all four CYP genes and these were prepared for heterologous expression in *S. cerevisiae*.

### Heterologous expression and functional analysis of CYP proteins

**Fig. 5:**
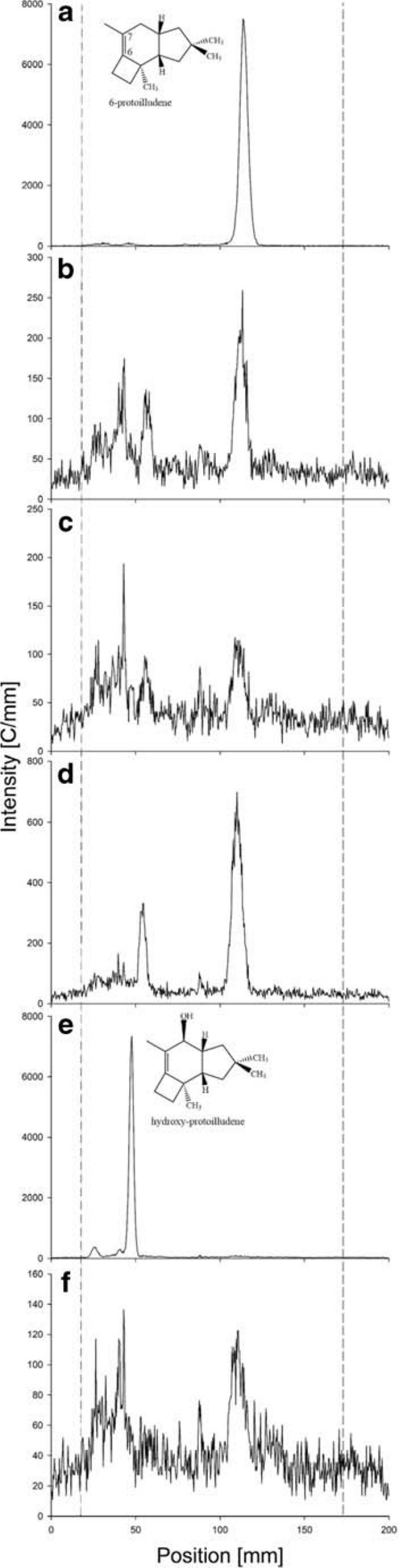
Radio-TLC chromatograms of feeding assays with ^3^H-labeled 6-protoilludene to confirm the activity of CYP-Arm3. The cDNAs corresponding to CYP-Arm1–4 were transferred to the yeast expression plasmid pYES-DEST52 and introduced into *Saccharomyces cerevisiae* CEN.PK2-1C cells. CYP gene expression and protein localization in the microsomal fraction were confirmed in each case by western blot, using antibodies against the His_6_ tag (Fig. S2). *Taxus chinensis* cytochrome P450 reductase (*Tc*CPR) was co-expressed in the same cells. (A) Substrate ^3^H-labeled protoilludene. (B) Negative control, *n*-pentane extract from CEN.PK2-1C cells expressing *Tc*CPR alone. (C) CEN.PK2-1C cells expressing *Tc*CPR and CYP-Arm1. (D) CEN.PK2-1C cells expressing *Tc*CPR and CYP-Arm2, showing minimal activity on the substrate. (E) CEN.PK2-1C cells expressing *Tc*CPR and CYP-Arm3, showing complete conversion of 6-protoilludene into a putative hydroxyprotoilludene. (F) CEN.PK2-1C cells expressing *Tc*CPR and CYP-Arm4

The intronless full-length cDNA sequences representing each CYP gene were separately transferred to an expression construct featuring the inducible *GAL1* promoter and an in-frame C-terminal His_6_ tag. Following the transformation of *S. cerevisiae* strain CEN.PK2-1C, heterologous expression was induced with galactose, the recombinant yeast cells were harvested, and the recombinant CYP proteins were recovered in the microsomal fractions. Western blot analysis using an antibody specific for the His_6_ tag confirmed the correct localization of the proteins and the anticipated molecular weight of 57–60 kDa (Fig. S2). For functional testing, the yeast cells were co-transformed with *Tc*CPR, which reduces the cytochrome P450 monooxygenases and thus boosts their catalytic activity (Jennewein et al. 2005), because earlier studies indicated that the endogenous level of CPR in *S. cerevisiae* is limiting (Pompon and Coon 1984). We fed the cells overnight with 15,000 cpm of [^3^H]-6-protoilludene per 5 mL of culture, and the organic extracts were analyzed by radio-TLC (Fig. 5). The analysis of CEN.PK2-1C cells expressing CYP-Arm3 plus *Tc*CPR indicated the near complete conversion of [^3^H]-6-protoilludene to a more polar, apparently hydroxylated product. A similar result was observed for CYP-Arm2, although the conversion was much less efficient. Interestingly, the re-extraction of 6-protoilludene from the yeast cell culture using *n*-pentane was challenging but seemed to improve significantly for the hydroxylated derivatives.

**Fig. 6:**
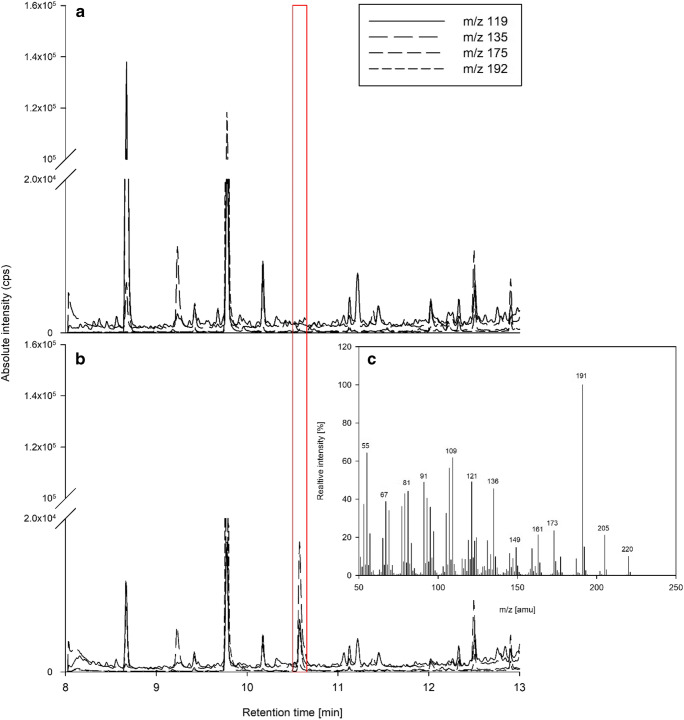
Nonradioactive feeding assay and GC/MS analysis of extracts from cells supplemented with exogenous 6-protoilludene. GC/MS chromatogram and analysis by selective ion monitoring (SIM) based on literature values for the masses of 6-protoilludene (Joulain and König 1998) and 6-hydroxy-7-protoilludene (Nozoe et al. 1977). (A) Extract of negative control cells expressing *Tc*CPR alone. (B) Extract from cells expressing CYP-Arm3 and *Tc*CPR reveals an additional peak with a retention time of 10.6 min (red box). The mass spectrum (C) indicates that the molecule is a putative hydroxyprotoilludene but not the anticipated 6-hydroxy-7-protoilludene. Exogenously fed 6-protoilludene substrate (with a retention time of 8.7 min) is markedly reduced within the extract of cells overexpressing the CYP-Arm3

For GC/MS analysis, the yeast cultures were incubated with unlabeled 6-protoilludene produced in vitro from farnesyl diphosphate using the recombinant protoilludene synthase. As above, the expression of the CYP proteins was induced in yeast cells co-expressing *Tc*CPR, and in vivo feeding was followed by extraction with *n*-pentane. GC/MS analysis of the fraction extracted from cells expressing CYP-Arm3 and *Tc*CPR revealed a peak that was not present in the control expressing *Tc*CPR alone (Fig. 6). The corresponding mass spectrum identified the product as a hydroxyprotoilludene, but the fragmentation pattern differed significantly from the previously reported profile of 6-hydroxy-7-protoilludene (Nozoe et al. 1977).

### Total biosynthesis of hydroxyprotoilludene by recombinant *S. cerevisiae*

**Fig. 7:**
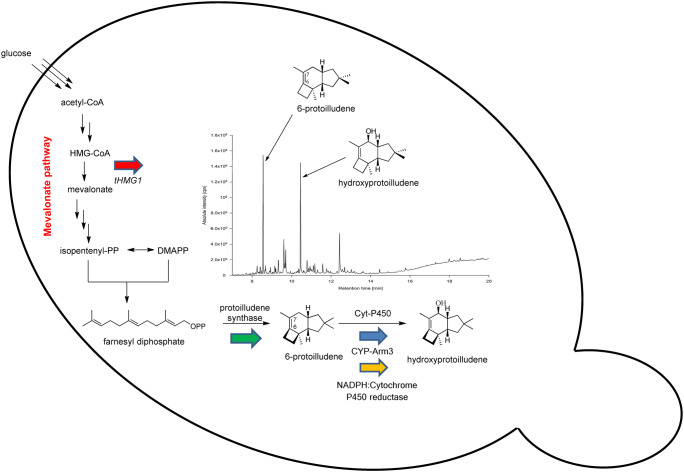
The production of a novel hydroxyprotoilludene in *Saccharomyces cerevisiae* by metabolic engineering. The cells were engineered to overexpress the catalytic domain of 3-hydroxy-3-methylglutaryl-CoA-reductase (red arrow) to increase the production of farnesyl diphosphate (FPP), as well as protoilludene synthase (green arrow), CYP-Arm3 (blue arrow), and *Tc*CPR (yellow arrow). GC analysis of the extract from this strain revealed 6-protoilludene and hydroxyprotoilludene as the main metabolites

The novel hydroxyprotoilludene revealed by GC/MS was characterized de novo by NMR spectroscopy, but first, it was necessary to produce sufficient quantities (40 mg) of the pure compound. We therefore engineered a strain of *S. cerevisiae* capable of total biosynthesis of this product (Fig. 7) by transforming strain CEN.PK2-1C with episomal expression vectors containing *A. gallica CYP-Arm3*, *Tc*CPR, a truncated version of the yeast HMG-CoA reductase (tHMGR), and *A. gallica* protoilludene synthase (Engels et al. 2011). The truncated, deregulated HMG-CoA reductase was included to increase flux though the mevalonic acid pathway and thus provide additional farnesyl diphosphate (Dimster-Denk et al. 1994; Donald et al. 1997; Engels et al. 2008). Fermentation was carried out at the 3-L scale, with samples taken periodically for the analysis of heterologous protein accumulation (Fig. S3), and the product was purified from the clarified culture supernatant.

### Purification of hydroxyprotoilludene and structural analysis

The structure of the unknown hydroxyprotoilludene was determined by ^1^H and ^13^C NMR spectroscopy (Fig. S4), and the product was identified as 8α-hydroxy-6-protoilludene (Fig. S5). The complete ^1^H and ^13^C NMR shifts and their assignments based on 2D NMR spectra are shown in Table 1. The assignments were consistent with the structures of other known melleolides or armillyl orsellinates, but the identification of 8α-hydroxy-6-protoilludene was confirmed by acquiring HMBC and NOESY spectra (Figs. S6 and S7). Both the hydrogen atom and the hydroxyl group at position 8 were bounded on C-8 (74.77 ppm). Starting in the HMBC spectrum from methyl group 13, couplings were observed to C-6, C-7, and C-8. The stereochemistry of the compound was confirmed by the observed nuclear Overhauser effects (NOEs) in the NOESY spectrum. NOEs were clearly observed between H-8 and H-10_B_ and methyl group 1 at position 2, which confirmed that these atoms are located on the same side of the molecule.

**Table 1:** NMR analysis of 8α-hydroxy-6-protoilludene: ^1^H and ^13^C chemical shifts (δ in ppm), coupling constants (Hz) and NOE cross peaks in relation to CHDCl_2_ (^1^H 5.32 ppm, ^13^C 54.00 ppm). Abbreviations: s/S, singlet; d/D, doublet; t/T, triplet; Q, quartet; dd, doublet of doublets; dt, doublet of triplets; m, multiplet. *Uncertain assignment

Number	^13^C-signal	^1^H-signal	^*n*^*J* coupling constant	NOE signal
1_A_ 1_B_	41.74 (T)	1.38 (ddd) 1.32 (dd)	^2^*J*_1A,1B_ = 12.5; ^3^*J*_1A,2_ = 8.0 ^3^*J*_1B,2_ = 10.8; ^4^*J*_1A,10A_ = 2.0	1_A_-15 1_B_-12
2	47.07 (D)	2.34 (dt)	^3^*J*_2,1A_ = 8.0; ^3^*J*_2,1B_ = 10.8 ^3^*J*_2,9_ = 12.0	2-4_A_; 2-9; 2-15
3	45.80 (S)	-		-
4_A,B_	36.74 (T)	1.81 (2H, m)	^2^*J*_4A,4B_ = 9.2 ^3^*J*_4,5_ = 10.6 and 3.8*	4-2; 4-12
5_A_ 5_B_	25.20 (T)	2.51 (dddq) 2.70 (dddq)	^2^*J*_5A,5B_ = 14.8; ^5^*J*_5,13_ = 2.5* ^3^*J*_5A,4_ = 7.2 and 1.2* ^3^*J*_5B,4_ = 11.1 and 3.8	5_A_-5_B_; 5_A_-4; 5_A_-13 5_B_-4; 5_B_-12
6	141.79 (S)	-		-
7	127.10 (S)	-		-
8	74.77 (D)	3.94 (d, broad)	^3^*J*_8,9_ = 8.9; ^4^*J*_8,13_ = 1.3*	8-OH; 8-10_B_; 8-12
9	51.21 (D)	2.18 (m)	^3^*J*_9,2_ = 12.0; ^3^*J*_9,10A_ = 7.4 ^3^*J*_9,8_ = 8.9; ^3^*J*_9,10B_ = 10.6	9-2; 9-15
10_A_ 10_B_	47.17 (T)	1.72 (ddd) 1.12 (dd)	^2^*J*_10A,10B_ = 12.1; ^3^*J*_10A,9_ = 7.5 ^4^*J*_10A,1A_ = 2.0; ^3^*J*_10B,9_ = 10.6	10_A_-10_B_; 10_A_-9; 10_A_-15 10_B_-8
11	40.17 (S)	-		-
12	20.63 (Q)	1.02 (3H, s)		12-1_B_; 12-4; 12-5_B_; 12-8
13	11.39 (Q)	1.60 (3H, dt)	^4^*J*_13,5_ = 1.3*; ^4^*J*_13,8_ = 2.4*	13-5_A_
14	29.88 (Q)	1.08 (3H, s)		14-1_B_
15	27.40 (Q)	0.95 (3H, s)		15-1_A_; 15-2; 15-9; 15-10_A_
–OH	-	1.44 (s, broad)		OH-8

## Discussion

Protoilludene-type aryl esters are bioactive secondary metabolites produced solely by fungi of the genus *Armillaria*. Many of these natural products are potentially useful as drug leads. For example, some lack broad cytotoxicity but selectively kill tumor cells and could be developed into new chemotherapeutic compounds for systemic administration (Chen and Chen 2014). Others are generally cytotoxic and would cause systemic off-target effects but could potentially be used for targeted therapy when combined with antibodies or other ligands. Recently, it was discovered that melleolides bearing an α,β-unsaturated aldehyde group actively inhibit 5-lipoxygenase activity, the keg enzyme in pro-inflammatory leukotriene biosynthesis (König et al. 2019). Some melleolides show antifungal activity against common agricultural pathogens. For example, armillane, 10α-hydroxyarmillarin, and 4-*O*-methylarmillaridin are effective against *Phytophthora cinnamomi*, which is responsible for root rot or die back disease in many crops (Donnelly and Hutchinson 1990). Pathogenic fungi are often overlooked as a threat to human health, but this could change as previously isolated fungi begin to colonize formerly inaccessible regions due to the transformation of local climates (Bills and Gloer 2016; Casadevall 2017; Fones et al. 2017).

The honey mushroom *A. gallica* produces a diverse range of melleolides, but little is known about the biosynthesis of these compounds. We have identified a melleolide gene cluster in this species which is similar to one within *A. mellea* (Wick et al. 2016) and determined the functions of a monooxygenase catalyzing early reactions in the pathway. The potent cytotoxic and antimicrobial activities of melleolides prompted us to isolate the *A. gallica Pro1* gene encoding 6-protoilludene synthase, the pathway-committing enzyme (Engels et al. 2011). Subsequent genome walking indicated that *Pro1* is nestled within a gene cluster encoding four CYP proteins, a GMC oxidoreductase, and a methyltransferase. Functional testing of cloned CYP cDNA sequences using [^3^H]-6-protoilludene as a substrate confirmed the activities of two of the enzymes. In vivo feeding assays in yeast surprisingly revealed that biosynthesis proceeds through an 8α-hydroxy-6-protoilludene intermediate produced by CYP-Arm3, rather than 6-hydroxy-7-protoilludene. A yeast strain was engineered to produce the novel melleolide intermediate in sufficient quantities for structural analysis by NMR spectroscopy.

**Fig. 8:**
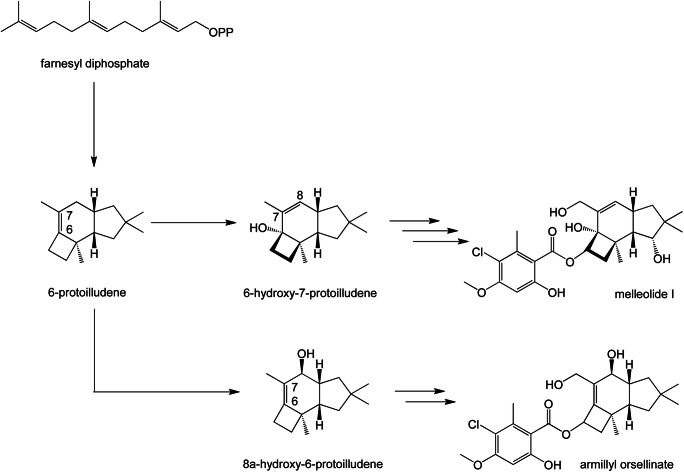
The novel hydroxyprotoilludene was identified as 8α-hydroxy-6-protoilludene by NMR spectroscopy. This is the first step in the biosynthesis of melleolides with the carbon double bond in the 6,7 position (like armillyl orsellinate). The cytochrome P450 monooxygenase that converts this intermediate into 6-hydroxy-7-protoilludene; the first step towards the major metabolites in *A. gallica* (e.g., melleolide I) is still unknown

The activity of CYP-Arm2 appeared to produce small amounts of a more hydrophilic product when fed with the 6-protoilludene substrate, suggesting opportunistic substrate catalysis. The subsequent hydroxylation steps are now under investigation. The enzymes CYP-Arm1 and CYP-Arm4 did not produce novel products when fed with 6-protoilludene, suggesting that they require different substrates and may perform downstream modification steps. The melleolide biosynthesis pathway may therefore branch after the farnesyl diphosphate cyclization step, leading to diverse terminal melleolide polyketide products (Fig. 8). In addition, the gene directly responsible for 6-hydroxy-7-protoilludene synthesis has yet to be identified, perhaps indicating the presence of a multi-cluster biosynthesis pathway similar to the illudin pathway in *Omphalotus olearius* (Wawrzyn et al. 2012). Therefore, we are currently screening the *A. gallica* genome to identify further gene clusters containing melleolide biosynthesis genes. Once isolated and expressed, these sequences will be tested against diverse substrates, starting with 8α-hydroxy-6-protoilludene produced by CYP-Arm3, and later with more advanced intermediates to identify the precise catalytic activity and position within the melleolide biosynthesis pathway.

## Supplementary information

ESM 1 (PDF 1137 kb)
