# Supplementary material for: Isolation of a gene cluster from Armillaria gallica for the synthesis of armillyl orsellinate–type sesquiterpenoids
Source: Appl Microbiol Biotechnol. 2020 Nov 16;105(1):211–24. doi: 10.1007/s00253-020-11006-y (PMC7778616; doi:10.1007/s00253-020-11006-y)
Supplement: Supplementary file 1 — (PDF 1137 kb) [file 253_2020_11006_MOESM1_ESM.pdf]

Supplementary Material:

Applied Microbiology and Biotechnology

Isolation of a gene cluster from *Armillaria gallica* for the synthesis of armillyl orsellinate-type sesquiterpenoids

Benedikt Engels[1,2] · Uwe Heinig[1,3] · Christopher McElroy[1] · Reinhard Meusinger[4] · Torsten Grothe[5] · Marc Stadler[6] · Stefan Jennewein[1*]

[1] Fraunhofer Institute for Molecular Biology and Applied Ecology, Forckenbeckstrasse 6, 52074 Aachen, Germany.

[2] Present address: Jennewein Biotechnologie GmbH, Maarweg 32, Rheinbreitbach, Germany.

[3] Present address: Department of Plant & Environmental Sciences, Weizmann Institute of Science, P.O. Box 26, 7610001 Rehovot, Israel.

[4] Clemens Schöpf Institute of Organic Chemistry and Biochemistry, Technical University of Darmstadt, 64287 Darmstadt, Germany.

[5] Mibelle Group Biochemistry, Bolimattstrasse 1, 5033 Buchs, Switzerland.

[6] Department of Microbial Drugs, Helmholtz Centre for Infection Research, Inhoffenstrasse 7, 38124 Braunschweig, Germany.

* To whom correspondence should be addressed: Fraunhofer Institute for Molecular Biology and Applied Ecology, Forckenbeckstrasse 6, 52074 Aachen, Germany; email: stefan.jennewein@ime.fraunhofer.de; phone: +49 241 6085 12121; fax: +49 241 6085 10000

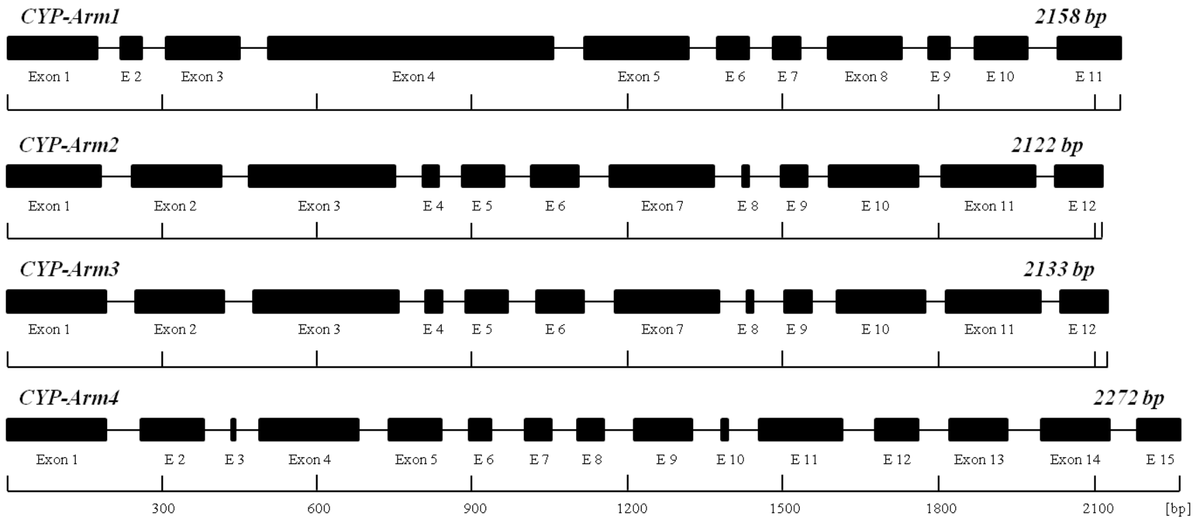

Figure S1. Schematic representation of the *A. gallica* cytochrome P450 monooxygenase genes *CYP-Arm1*, *CYP-Arm2*, *CYP-Arm3* and *CYP-Arm4*. Coding sequences (exons) are shown as black boxes and intervening introns as lines. *CYP-Arm2* and *CYP-Arm3* have an identical intron–exon structure. *CYP-Arm4* contains the most exons, one of which (exon 3) is only 4 bp in length.

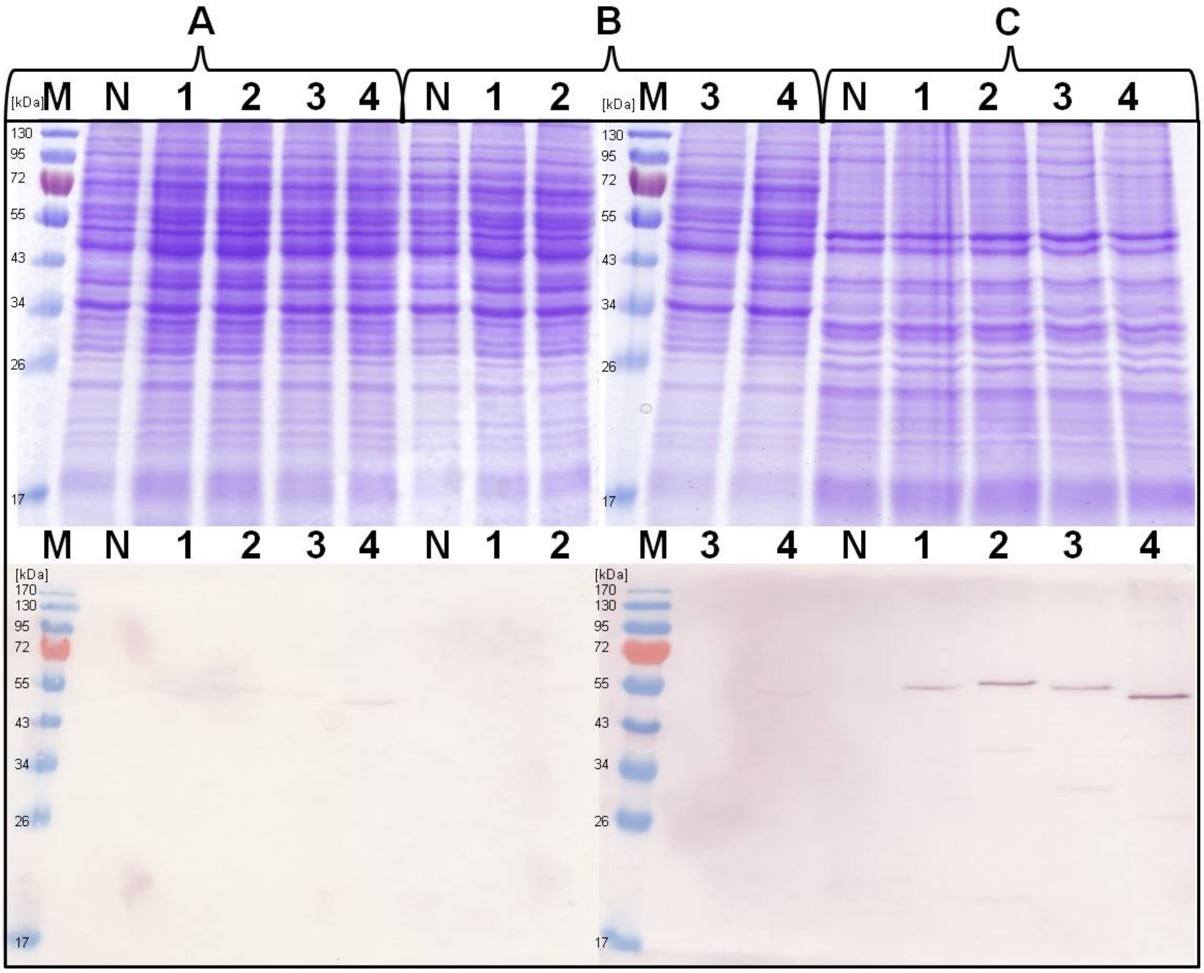

Figure S2. SDS PAGE (upper) and Western blot (lower) analysis of microsomal fractions from *Saccharomyces cerevisiae* clones expressing CYP-Arm1 (1), CYP-Arm2 (2), CYP-Arm3 (3) or CYP-Arm4 (4), compared with a negative control clone (N). (A) Direct protein extract post cell lysis. (B) Ultracentrifugation supernatant samples acquired in preparation of microsomal extract. (C) Correct localization of the proteins in the microsomal protein fraction and confirmation of the anticipated molecular weights.

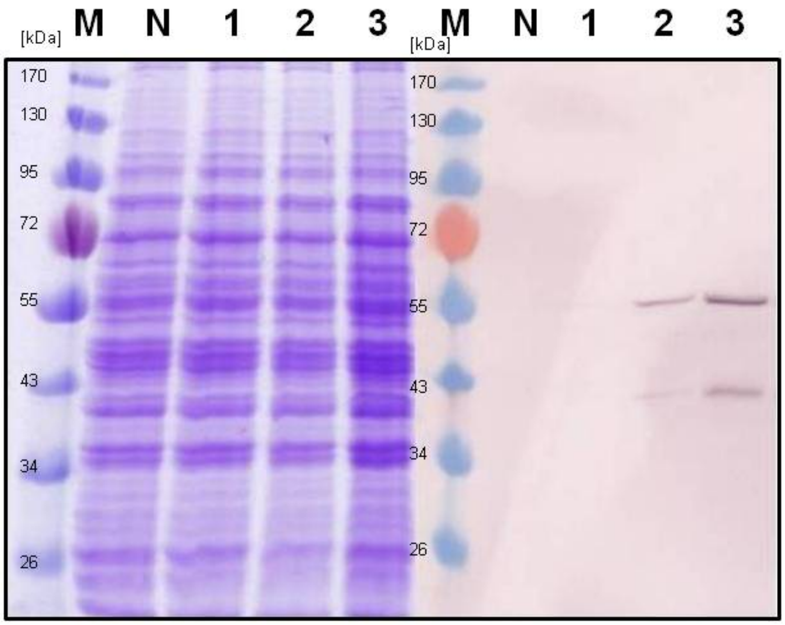

Figure S3. Western blot analysis of His_6-tagged protoilludene synthase (40 kDa) and CYP-Arm3 (55 kDa) reveals stable expression in fermentation samples during the heterologous production of hydroxyprotoilludene. M = protein ladder; N = negative control; 1 = induction time point – addition of galactose; 2 = 2 h post-induction; 3 = 4 h post-induction. 2 mL fermentation samples lysed and clarified prior to 20µg of lysate loaded per lane.

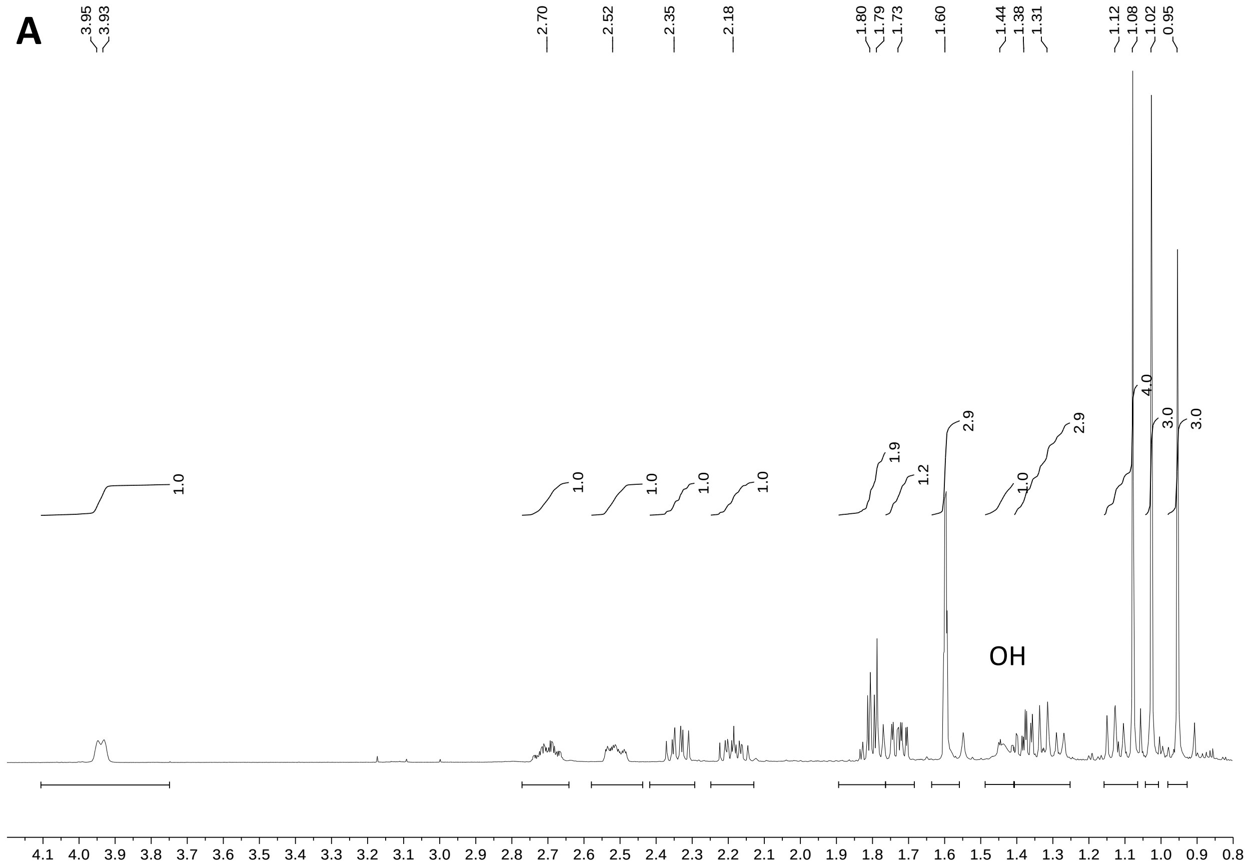
A
3.95
3.93
2.70
2.52
2.35
2.18
1.80
1.79
1.73
1.60
1.44
1.38
1.31
1.12
1.08
1.02
0.95
1.0
1.0
1.0
1.0
1.0
1.9
1.2
2.9
1.0
2.9
4.0
3.0
3.0
OH
4.1 4.0 3.9 3.8 3.7 3.6 3.5 3.4 3.3 3.2 3.1 3.0 2.9 2.8 2.7 2.6 2.5 2.4 2.3 2.2 2.1 2.0 1.9 1.8 1.7 1.6 1.5 1.4 1.3 1.2 1.1 1.0 0.9 0.8

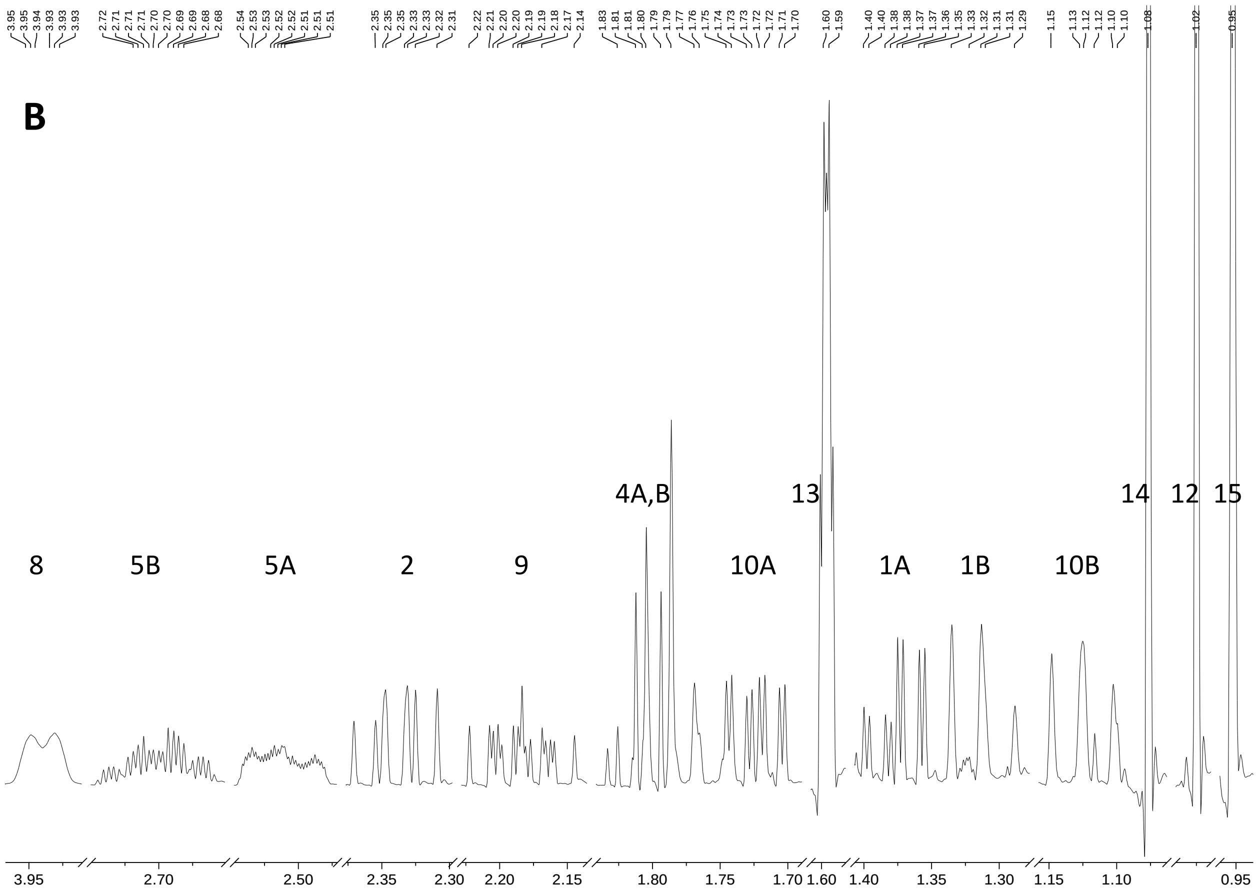
B
8
5B
5A
2
9
4A,B
10A
13
1A
1B
10B
14
12
15
3.95
2.70
2.50
2.35
2.30
2.20
2.15
1.80
1.75
1.70
1.60
1.40
1.35
1.30
1.15
1.10
0.95

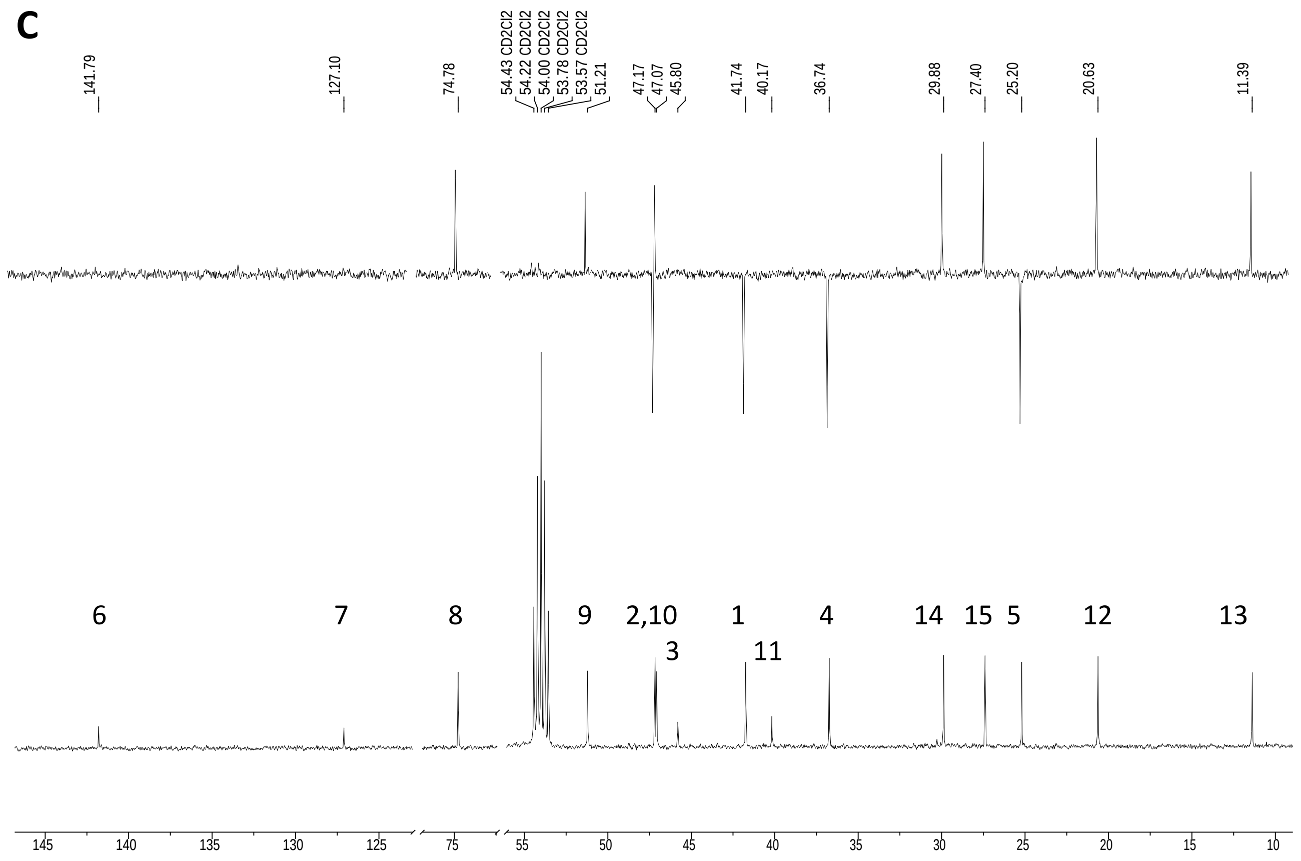

Figure S4. NMR spectra for the structural evaluation of the unknown hydroxyprotoilludene product, identifying it as 8α-hydroxy-6-protoilludene. (A) ^{1}H NMR spectrum with integral identification. (B) ^{13}C NMR spectrum. (C) ^{13}C-DEPT_135a NMR spectrum.

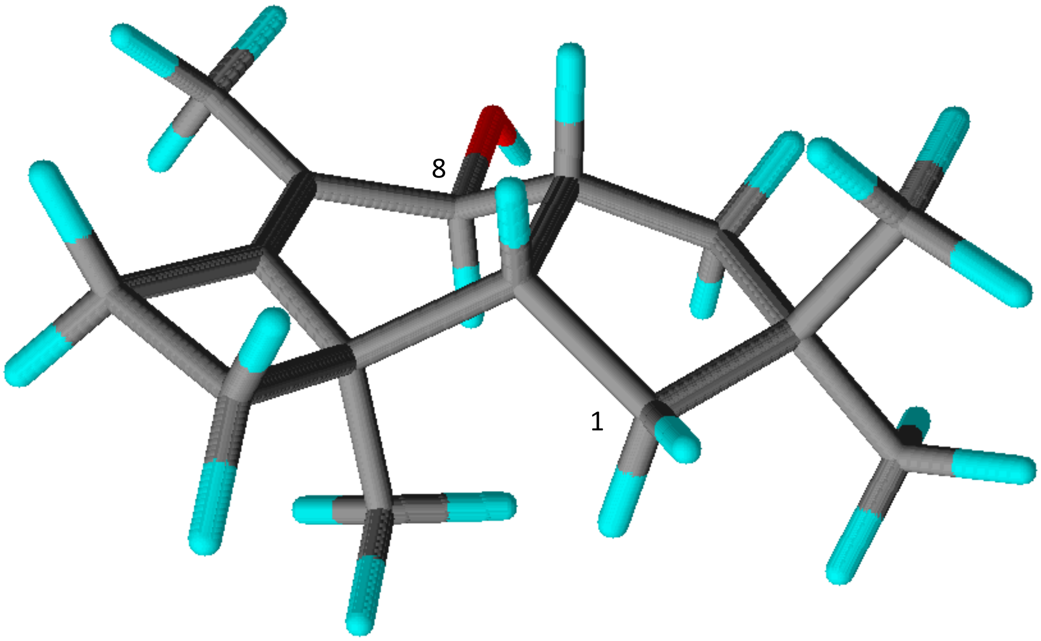

Figure S5. Molecular structure of 8α-hydroxy-6-protoilludene.

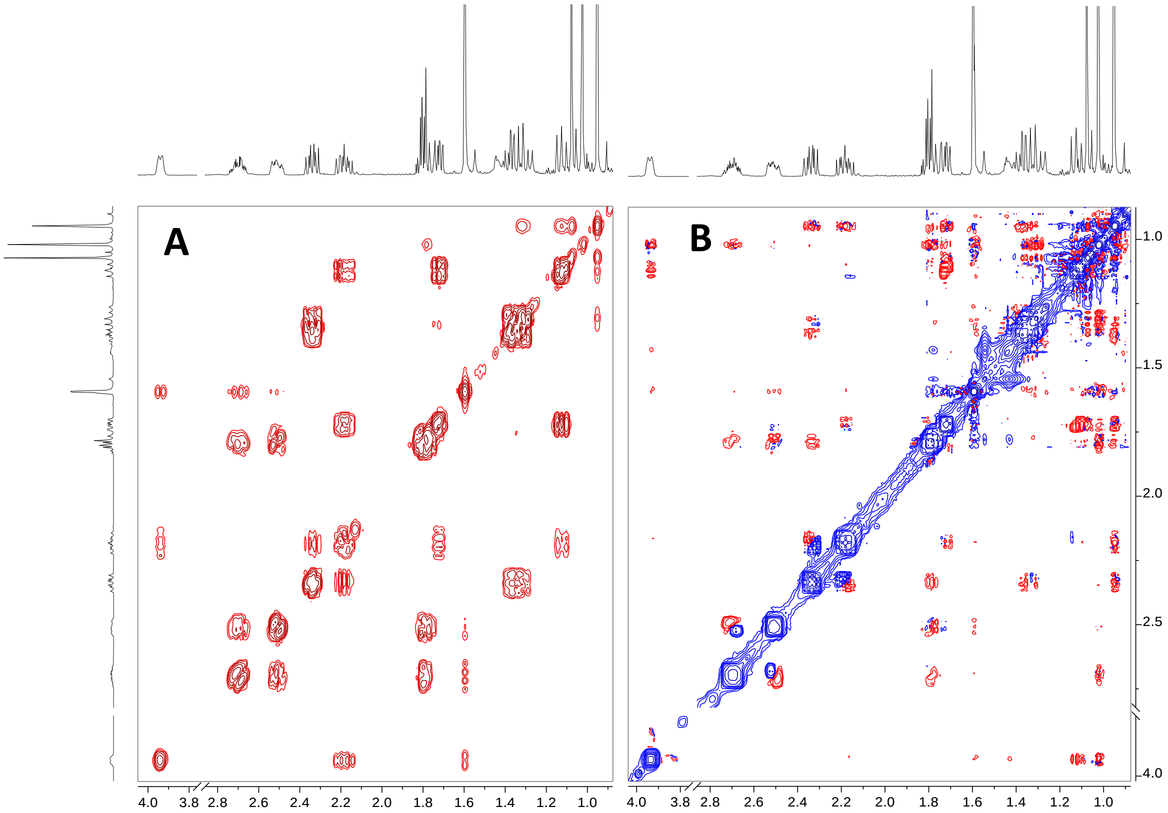

Figure S6. Two-dimensional NMR spectra for 8α-hydroxy-6-protoilludene. (A) gs-COSYDF spectrum. (B) gs-NOESY spectrum.

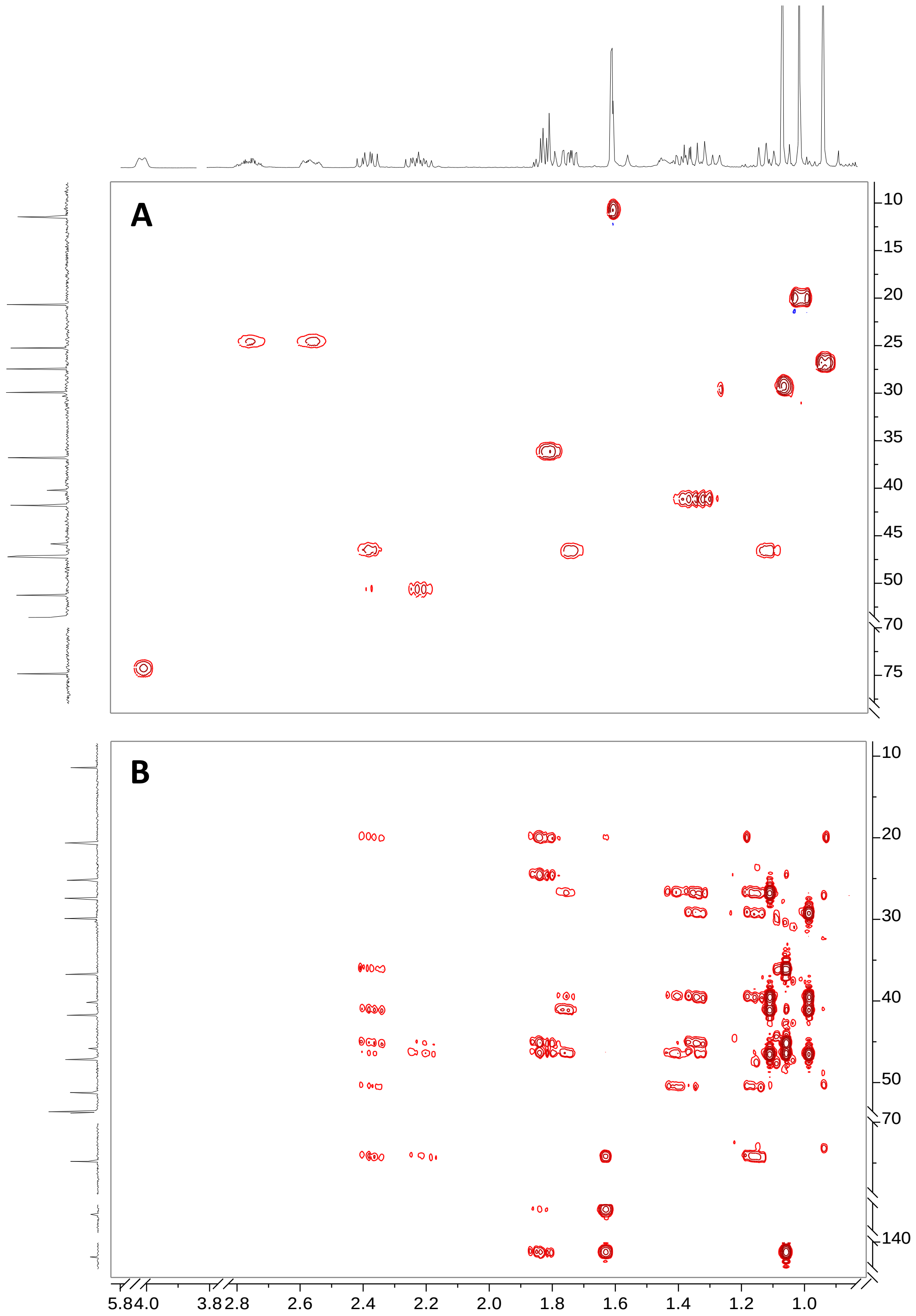

Figure S7. Heteronuclear NMR spectra for 8α-hydroxy-6-protoilludene. (A) gs-HSQC spectrum. (B) gs-HMBC spectrum.

Table S1 PCR primers for used for the generation of GenBank accession number MT277003.

Primer name	Sequence
5-1 T3 P1-1 for	GGCATACCAGCCTTCTGCC
5-1 T3 P1-1b for	GATGCGCTTGATTGATGGGTC
5-1 T3 P1-2 for	GCAGACGATGCGTGTGTCATC
5-1 T3 P1-3 for	CCTGTAATACGCGTCCTTCTC
5-1 T3 P1-4 for	CCCGTCGGCAAGCGAGTCGG
5-1 T3 P1-5 for	CGATTAGAGGTGAGCTGGCTG
5-1 T3 P1-6 for	CCTCGGTGCCGACATAGAAAG
5-1 T3 P1-7 for	CGACGTTGCAAACGCACACATG
5-1 S1 P2-1 for	CGTTGCGAAGCGGAAGTGC
5-1 S1 P2-1 rev	GAGTTACGCCAAGGTCGCGC
5-1 S1 P2-2 for	CTGAGGGCGAGATGTTCGTC
5-1 S1 P2-3 for	GAGATGTTTTCCGTATAGGATG
5-1 S1 P2-4 for	GGTGCTGATACGGTACAATTC
5-1 S1 P2-5 for	GGATGACTGAAGTTGGCTGGG
5-1 S1 P2-6 for	CCACCTCGGACCTCCAAACTTG
5-1 S1 P2-7 for	GGCCGTGAACAGGCTTCACC
5-1 S2 P3-1 for	CGTGGTACGTTCGGCTGTTG
5-1 S2 P3-1 rev	CATCTACTTGTCATCTACAC
5-1 S2 P3-2 for	GCAAGTCTCATCACCGGTATGC
5-1 S2 P3-3 for	GAGCCGATGAGATGGCAAATG

5-1 S2 P3-4 for	GGGATGTGTAAGGTCCTTCCAC
5-1 S2 P3-5 for	GCAGAACTCGATGCTGTCGTTG
5-1 S2 P3-6 for	GTAGAGTGTACTCCGCTGTAG
5-1 S2 P3-7 for	CCTAGCAAATACCTACGCCG
5-1 T7 P4-1 for	CTTTGACCAATCGACACTAG
5-1 T7 P4-1b for	GCTGAGTCAAGCCGATGCAG
5-1 T7 P4-2 for	CTACAGGTCGGTGTGATAGAGG
5-1 T7 P4-3 for	CGGTAACAAGACACTGCTGG
5-1 T7 P4-4 for	GACATCATAGAGTCGCCTGGG
5-1 T7 P4-5 for	CACTTGCATCTGGCTGCCTGC
5-1 T7 P4-6 for	CCATAGGCGTGTATCGTCCG
5-1 T7 P4-7 for	CTCTGCCAGAACCCATACTCTG
27-2 T3 P1-1 for	GAGACAAGGATGACAACGATACTC
27-2 T3 P1-2 for	GTCAGCGGCATCCGAATCTGACG
27-2 T3 P1-3 for	GACCGAGAGGCCTTCCTCTCATC
27-2 T3 P1-4 for	GTACGTGCACAACCATTGAATCC
27-2 T3 P1-5 for	GACATAGCAATGATGCTTTGGGC
27-2 T3 P1-6 for	CTTAACAGGTTCTGGGCTCCCGTTG
27-2 T3 P1-7 for	CCATCTTCCGCAATCTGCGAGCAG
27-2 T3 P1-7b for	GTGAAGGAGGGCTATTGTAGGGTG
27-2 T3 P1-7 rev	CGTCGTTAGATGTCGGGGTCTCG
27-2 T3 P1-8 for	CAGCGGTATCGTCTTTGTACCTG

27-2 T3 P1-8b for	GCACCCGAAGATACCACAAAGACG
27-2 T3 P1-9 for	CATGTAGCACTCTCGCCATCCTCGC
27-2 T3 P1-10 for	GTAGGGATAAGTTACGAGCCTTGAG
27-2 T3 P1-11 for	CACTACAATTCCCGTGGAGTTTTCTG
27-2 T3 P1-12 for	CACATCCTGTCTCCACTTCAAGCCGAG
27-2 T3 P1-13 for	GTCGGTGAATATCAGTTTCAATCTC
27-2 T3 P1-14 for	CAAGGTGGATGCTACGTGCGTTATC
27-2 T7 P2-1 for	CCTTGACGGCTTATGGGAAGAG
27-2 T7 P2-2 for	CCTACGACGAGCTTCACGAAGG
27-2 T7 P2-3 for	CGATATACGGTATCAATTCGGGTGC
27-2 T7 P2-4 for	GGTACCGATTAGGTGTGTACAGC
27-2 T7 P2-5 for	GCCATCAGTGGGCACGCACTGATG
27-2 T7 P1-5b for	CAAGCTACAAGAAGGTATGTGATCC
27-2 T7 P2-6 for	GACGCAAAGTTCGACGAGATGTCGAC
27-2 T7 P2-7 for	GCACAGTTACCAACCGCGATGCAAG
27-2 T7 P2-8 for	CTCCTGTATCACCATTCGTATCTC
27-2 T7 P2-9 for	CGCCGATCTCTTTGGTGAACTGGATG
27-2 T7 P2-10 for	CGTGGGATATATCTGCGCTTTGCG
27-2 T7 P2-11 for	CAACGCATCATGTGTCGTCTTTCAGTGG
27-2 T7 P2-12 for	GCTCACCGCCCTTCACGAATTAGCTC
27-2 T7 P2-13 for	CGGACACTCTTTAATACTCCTCGTTC
27-2 T7 P2-14 for	CGAAGTGGATTTCGAAGTTGAGCTAC

27-2 T7 P2-15 for	GCATAGATGCTGCGTTACCTTCTGC
25-1 T3 P1-1 for	GTCAACGTGGATGGGAATGATGATG
25-1 T3 P1-2 for	CAGGAGTTTGATGGTGAAGCCTAGC
25-1 T3 P1-3 for	GTGATACTTGCCCTTTAGATCTCG
25-1 T3 P1-4 for	CGGTAGCACATCCTCCATTTATAACG
25-1 T3 P1-5 for	GAAGGGAGGATTTATCTGGAGGGC
25-1 T3 P1-6 for	GAATGGTTGCGACAGATTTGACGC
25-1 T3 P1-7 for	GTATGTAGGTGGTACAGTATTGTAGACAG
25-1 T3 P1-8 for	CCATGGAGCCGTTTTGCCAGGTAGG
25-1 T3 P1-8 rev	CATCAGTATAATCCTGTCCCTCAGC
25-1 T3 P1-9 for	CATGTGGTGACCTCCTCCTTGAACGCTG
25-1 T3 P1-10 for	CAATACTGCCAAGGCAGTTGTCACCAG
25-1 T3 P1-11 for	GCTGATGAACCCAAGCGGATTAGAGAGG
25-1 T3 P1-12 for	GCTCTGTCGGCGCTATTGCCTTTC
25-1 T3 P1-13 for	CTCTGTGGGGGAGTATGGGAAG
25-1 T3 P1-14 for	CTTCAAGATGCGGCCATTCTG
25-1 T3 P1-14 rev	CTTCATGTGATGCACCATGATAGC
25-1 T3 P1-15 for	GCTGAGTTTGGAGTAGACAGATTG
25-1 T7 P2-1 for	CACGCAGGAGTTCTTCATATGTAATG
25-1 T7 P2-2 for	GCAGCAATTGTCAGTGGTTATCACAG
25-1 T7 P2-3 for	GTTGCCTGATATATCGACGTGGGAACG
25-1 T7 P2-4 for	GCCTGTTGAGTTCGAAATTCTAACG

25-1 T7 P2-5 for	GTACCGTACGTACATTGATTCATG
25-1 T7 P2-6 for	CGAATTGGCGTAACGCTGACCATG
25-1 T7 P2-7 for	GAATTGGGACATCTTGCCACCACTC
25-1 T7 P2-8 for	GGCTACAATGACTGGAATAAAATGCG
25-1 T7 P2-9 for	GCCAAAGAGACCAGAATGGAAATGG
25-1 T7 P2-10 for	GAGTCTGTGAGTTCTACTTTGAGTGG
25-1 T7 P2-11 for	CTGTGGTCATTGAGAGATTGCTCACTC
25-1 T7 P2-12 for	CACAACTTGGAATCTCTCAAGGCTAAC
25-1 T7 P2-13 for	GCATCCAACAGAAAGCATGACAGCAC
25-1 T7 P2-14 for	CTGACTGTGATGGGGGAGATATCGG
25-1 T7 P2-15 for	CTGTCTCAGCAGCTTCGAGAAGG
25-1 T7 P2-15 rev	GTCTTTATTCTAGTCTCACGAGC

Genome walker PCR methods

Herculase II polymerase: 95°C 5min, (95°C 30s; 65°C*30s; 72°C 5min) x 30 cycles, 72°C 10min

*or alternative annealing temperature for primer pairs
